# The Sediment Test for Milk

**Published:** 1914-08

**Authors:** 


**The Sediment Test for Milk.** Way, in the Cleveland Medical Journal, 1914, No. 5, describes a classification of milk according to the dirt resulting from the filtration of ½ pint through a filter consisting of an absorbent cotton disc, free from sizing, about one-eighth of an inch in diameter.—
C. G. L-W.

